# The alcohol use disorders identification test (AUDIT): validation of a Nepali version for the detection of alcohol use disorders and hazardous drinking in medical settings

**DOI:** 10.1186/1747-597X-7-42

**Published:** 2012-10-05

**Authors:** Bickram Pradhan, François Chappuis, Dharanidhar Baral, Prahlad Karki, Suman Rijal, Antoine Hadengue, Pascal Gache

**Affiliations:** 1 Department of Internal Medicine, B.P Koirala Institute of Health Sciences, Dharan, 56700, Nepal; 2 Division of International and Humanitarian Medicine, Geneva University Hospitals, Rue Gabrielle-Perret-Gentil 4, 1211, Geneva, 14, Switzerland; 3 School of Public health, B.P Koirala Institute of Health Sciences, Dharan, 56700, Nepal; 4 Rue des deux-ponts 20, Geneva, 1205, Switzerland

**Keywords:** Alcohol use disorder, AUDIT, SCID for DSM- IV

## Abstract

**Background:**

Alcohol problems are a major health issue in Nepal and remain under diagnosed. Increase in consumption are due to many factors, including advertising, pricing and availability, but accurate information is lacking on the prevalence of current alcohol use disorders. The AUDIT (Alcohol Use Disorder Identification Test) questionnaire developed by WHO identifies individuals along the full spectrum of alcohol misuse and hence provides an opportunity for early intervention in non-specialty settings. This study aims to validate a Nepali version of AUDIT among patients attending a university hospital and assess the prevalence of alcohol use disorders along the full spectrum of alcohol misuse.

**Methods:**

This cross-sectional study was conducted in patients attending the medicine out-patient department of a university hospital. DSM-IV diagnostic categories (alcohol abuse and alcohol dependence) were used as the gold standard to calculate the diagnostic parameters of the AUDIT. Hazardous drinking was defined as self reported consumption of ≥21 standard drink units per week for males and ≥14 standard drink units per week for females.

**Results:**

A total of 1068 individuals successfully completed the study. According to DSM-IV, drinkers were classified as follows: No alcohol problem (n=562; 59.5%), alcohol abusers (n= 78; 8.3%) and alcohol dependent (n=304; 32.2%). The prevalence of hazardous drinker was 67.1%. The Nepali version of AUDIT is a reliable and valid screening tool to identify individuals with alcohol use disorders in the Nepalese population. AUDIT showed a good capacity to discriminate dependent patients (with AUDIT ≥11 for both the gender) and hazardous drinkers (with AUDIT ≥5 for males and ≥4 for females). For alcohol dependence/abuse the cut off values was ≥9 for both males and females.

**Conclusion:**

The AUDIT questionnaire is a good screening instrument for detecting alcohol use disorders in patients attending a university hospital. This study also reveals a very high prevalence of alcohol use disorders in Nepal.

## Background

Alcohol use disorders (AUDs), which includes alcohol abuse and alcohol dependence are regarded as one of the most important public health problems [1,2]. AUDS are among the most prevalent mental disorders worldwide and rank high as a cause of disability burden in most regions of the world [3]. AUDs are less frequent, but still substantial in developing countries [4]. Nepal is a landlocked country in South Asia bordered by China and India with a population of approximately 30 million. Hinduism is practiced by a large majority of people in Nepal. Increase in alcohol consumption in this country is due to many factors which include advertising, affordability and availability. In most parts of the country liquor is freely available. The production of homemade form of alcohol for domestic use is allowed by the Liquor Control Act of Nepal, although much is sold in the market [5]. Types of traditional and local alcoholic beverages include country liquors (low quality alcohol made from molasses and produced in small distilleries), homemade liquors, *Jand* (made of rice), *Chang* (made of rice by a different method) and *Raksi* (home-brewed alcohol made out of rice, millet or barley). In rural Nepal, most traditional users of alcohol consume *Jand* as food. Locally produced alcoholic beverages tend to be cheap, which promotes accessibility and consumption.

Numerous studies have demonstrated that problem drinkers (hazardous drinking, alcohol abuse and alcohol dependence) can benefit from physician intervention [6], but lack of recognition of alcohol related problems by primary health care workers has been frequently reported [7,8]. Detection of alcohol abuse is particularly challenging in the busy out-patient and emergency departments. To identify patients with alcohol related problems in clinical and primary care settings, researchers have developed several screening questionnaires like CAGE and Michigan Alcoholism Screening Test (MAST). However CAGE identifies patient with alcohol problem but cannot distinguish between abuse and dependence. MAST contains 24 questions, which is not well suited in out-patient settings because of its length. In this context, the Alcohol Use Disorder Identification Test (AUDIT) was developed by the WHO [9]. Unlike other screening instruments, a definite advantage of the AUDIT is its capability to identify individuals along the full spectrum of problem drinking and hence providing an opportunity for early intervention in non-specialist settings [10,11]. The diagnostic performance of AUDIT has shown to be effective and compares favorably with other well known alcohol screening measurements [12-15]. AUDIT effectively identifies problem drinking in different countries and cultural groups [15-21]. Detection of AUDs, specifically alcohol abuse and dependence can be done in the busy out-patient departments by the AUDIT questionnaire, which allows early referral and intervention. Though it has been validated and found to be effective in different languages and cultural groups, it is yet to be adapted for use and validated in Nepal. The first validation of a Nepali version of AUDIT administered to a large sample of patients attending a university hospital in eastern Nepal is reported here. The reliability and diagnostic value of AUDIT was examined to identify individuals who meet DSM-IV criteria for alcohol dependence, alcohol dependence/abuse and the WHO definition of hazardous drinking.

## Methods

### Study site

The study was conducted at the B.P. Koirala Institute of Health Sciences (BPKIHS), which is a 700 bedded university hospital located in the terai plain of eastern Nepal.

### Patients

The recruitment of patients took place in the out-patient department of internal medicine between 15 March and 15 September 2009. Every fifth new (first consultation) patient attending MOPD was included. Inclusion criteria were patients aged 18 to 65 years of age, residency in Nepal, good understanding of Nepali language and signature of the informed consent sheet. Exclusion criteria were patients having cognitive disorders that could prevent them from answering the questionnaire adequately or had been previously diagnosed as having alcoholism.

### Description of the Nepali version of AUDIT

The 10-item AUDIT includes questions to assess alcohol intake (questions 1–3), alcohol dependence (questions 4–6) and alcohol-related problems (questions 7–10). Questions 1–8 are scored from 0 to 4, questions 9 and 10 are scored 0, 2 or 4, resulting in a maximum AUDIT score of 40. The English version of AUDIT was translated to Nepali by the following method. Two experts translated the original English questionnaire to Nepali. Two professional translators then independently translated the Nepali version back to English. Finally two investigators (BP and SR) discussed the differences and a consensus was reached for the final Nepali version.

### Operational definitions and reference standards

Alcohol dependence/abuse was the term used to define patients with either alcohol abuse or dependence. The Structured clinical interview diagnosis (SCID) for DSM-IV (Diagnostic and Statistical Manual of Mental Disorders, Fourth Edition) was used as the reference standard (gold standard) for alcohol dependence and alcohol abuse. The SCID for DSM-IV Axis I Disorders (SCID-I) is a semi-structured interview for making the major DSM-IV Axis I diagnoses [22]. Responses were given on a past twelve month’s basis. According to patient’s responses, they were classified into DSM-IV categories as alcohol abuse, alcohol dependence or absence of problem. Categories are exclusive for each other.

Hazardous drinking is defined as a quantity or pattern of alcohol consumption that places individuals at risk for adverse health events and is recognized by the WHO as a distinct disorder. It is defined as self reported consumption of ≥21 standard drink units (SDU) per week for males (equivalent to 210g ethanol), and ≥14 standard drink units per week (equivalent to 140g ethanol) for females [23]. One SDU amounts to 10g of pure ethanol [9,24]. Particular attention was devoted to quantify locally brewed beverages. Thus, several samples of locally brewed alcohols were analyzed for the alcohol concentration in the laboratory of the Hôpitaux Universitaires de Genève (Geneva University Hospitals) and the following results were obtained. Raksi 25%, Jand or Chhang 12% and Tongba 5.5%

### Procedures

The Institutional ethical review board of the university approved the study protocol and informed consents were obtained from the participants after the aims and objectives of the study had been explained. Two study nurses were trained by an addiction specialist from Switzerland (PG). Immediately after the patient agreed to participate and signed the informed consent, one nurse carried out the first stage interview. This consisted of helping the participants complete a self-report Nepali version of AUDIT questionnaire, demographic and clinical information. Later, the other study nurse, who was blinded to the results of the initial questionnaire, interviewed each participant about alcohol issues using SCID for DSM-IV. Interviewers also quantified alcohol intake of a typical week of the previous month.

Patients were asked in details about number of drinking days and the amount of alcohol consumed in the previous week. For assessment of alcohol intake colored photographs of different types of glasses and special type of containers used for consumption of locally brewed alcoholic beverages were used. If this week was untypical—to think about what they would drink on each day of a typical week. Then they were asked “Are there any days where you drunk more?” Alcohol consumption was measured by assessing quantity and frequency of alcohol use. Units of alcohol consumed per week were calculated by multiplying number of drinking days by unit of alcohol consumed per day and modified for extra drinks if required. Finally, patients were classified into three categories: no alcohol problem (abstinent or low-risk drinker), alcohol abuse (DSM-IV), or alcohol dependence (DSM-IV). The patients were also classified as hazardous drinkers with a self reported consumption of ≥21 SDU per week for males and ≥14 SDU per week for females. A pilot study was initially carried out in 50 patients to test logistics and provide additional training to the two study nurses. The results of the pilot phase were not included in the analysis.

### Data analysis

The internal consistency of the AUDIT was estimated by using Cronbach's α test. Cronbach’s α is a statistic calculated from the pair wise correlations between items. Internal consistency ranges between zero and one. A commonly-accepted rule of thumb is that an α of 0.6-0.7 indicates acceptable reliability, and 0.8 or higher indicates good reliability. High reliabilities (0.95 or higher) are not necessarily desirable, as this indicates that the items may be entirely redundant. The diagnostic parameters (sensitivity, specificity, positive and negative predictive values) of the AUDIT were separately calculated for alcohol dependence, alcohol dependence/abuse and hazardous drinking. DSM-IV diagnoses (alcohol abuse and dependence) and self reported alcohol consumption (hazardous drinking) were used as reference standard. Using SPSS 10.0 software, receiver operator characteristics (ROC) curves on the basis of a continuum of all possible values of total AUDIT scores were constructed. From the ROC curves the area under the curve (AUC) was used to assess the diagnostic capacity of the AUDIT. To assess criterion-related validity, sensitivity and specificity indices as well as the area under the receiver operating characteristic (ROC) curve were calculated. The best cut-off value was the one that maximized the sum of sensitivity and specificity. ROC curve allows the exploration of the entire range of sensitivities and specificities at each possible AUDIT cutoff score by showing sensitivity at the y axis and (1- specificity) at the y axis. Values of this area range from 0.5 to 1. A value of 1 indicates that the instrument gives a perfect discrimination between case and noncase, and a value of 0.5 implies an ability to discriminate no better than chance [25].

## Results

### Socio-demographic and alcohol-related data

1500 patients were assessed for eligibility, out of which 391 were excluded for various reasons (264 patients were above 65 years of age, 65 patients refused to participate, 43 had insufficient understanding of Nepali language and 19 were transferred to emergency ward)). Further 41 patients with incomplete file were excluded from the analysis. A total of 1068 individuals with age ranging from 18 to 65 years completed the evaluation and 124 of them were life time abstainers. The socio-demographic characteristics, alcohol consumption and alcohol-related diagnosis of the 944 patients with current alcohol consumption are presented in Table 1. There was a slight predominance of males (55%). The age was similar in both the sexes with mean age of 47.9 ± 11.8 in males and 47.5 ± 11.9 in females. 95.02% were married and Hinduism was the most prevalent religion (82.3%). Two-third of alcohol consumers were found to be daily drinkers. The AUDIT scores ranged from 0 to 38 with a median of 6. The median unit of alcohol consumed per week was similar for both males and females (28.4 and 28.0). According to DSM-IV, drinkers were classified as absence of alcohol problem (n=562; 59.5%), alcohol abuse (n=78; 8.3%) alcohol dependence (n=304; 32.2%) and alcohol abuse/ dependence (n=382; 40.5%). The prevalence of hazardous drinking was found to be 67.1%.

**Table 1:** Socio-demographic characteristics and alcohol diagnostic classification

	**Total (n= 944)**	**Male (n=497)**	**Female (n= 447)**
Age (mean ± SD)	47.7 ± 11.9	47.9 ± 11.8	47.5 ± 11.9
Marital status			
Married	897(95.02%)	469(94.4%)	428(95.8%)
Unmarried	32(3.4%)	23(4.6%)	9(2.01%)
Widowed/ divorced	15(1.6%)	7(1.4%)	8(1.8%)
Religion			
Hindu	777(82.3%)	412(82.9%)	365(81.7%)
Buddhist	165(17.5%)	83(16.7%)	82(18.3%)
Muslim	2(0.2%)	2(0.4%)	0
Occupation			
Housewife	221(23.4%)	0	221(49.5%)
Farmer	186(19.7%)	186(37.4%)	0
Unskilled worker	167(17.7%)	76(15.3%)	91(20.4%)
Student	75(7.9%)	35(7.1%)	40(8.9)
Military	58(6.1%)	58(11.7%)	0
Skilled worker	22(2.3%)	22(4.4%)	0
Government service	25(2.7%)	13 (2.6%)	12(2.7%)
Business	15(1.6%)	15(3.02%)	0
Unemployed	175(18.5%)	92(18.5%)	83(18.6%)
AUDIT score: median(IQR)	6(3–15)	7(4–16)	5(3–11)
Alcohol Intake in units/week: median (IQR)	28.0(14.0-52.9)	28.4(14.0-51.8)	28.0(14.0-53.9)
DSM IV classification:			
Abuse	78(8.3%)	50(10.1%)	28(6.3%)
Dependence	304(32.2%)	191(38.4%)	113(25.3%)
Dependence/abuse	382(40.5%)	241(48.5%)	141(31.5%)
Hazardous drinking*	633(67.1%)	293(58.9%)	340(76.1%)

* ≥ 21 units/week in males, ≥ 14 units/week in females.

SD= standard deviation.

IQR= inter quartile range.

### Internal consistency of the AUDIT

Cronbach's alpha coefficient for the AUDIT was 0.82, which indicate that the internal consistency level of the AUDIT was good. The inter item correlations were ≥ 0.6 in all questions except in question 3.

### Diagnostic validation of the AUDIT

**Figure 1:**
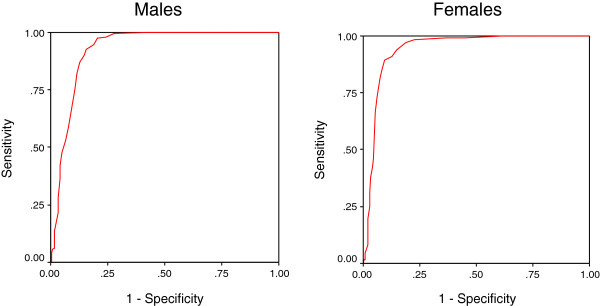
Receiver operating curve of the AUDIT using DSM-IV criteria for alcohol dependence.

**Figure 2:**
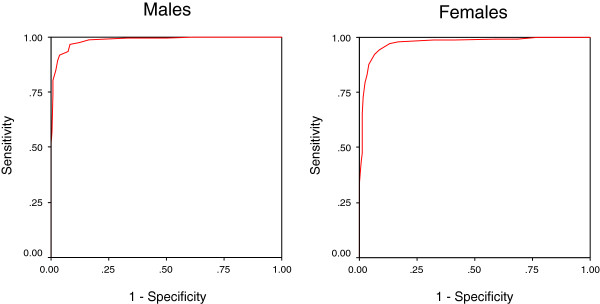
Receiver operating curve of the AUDIT using DSM-IV criteria for alcohol abuse/ dependence.

**Figure 3:**
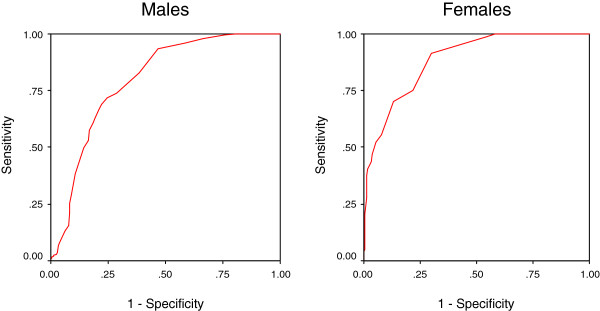
Receiver operating curve of the AUDIT using WHO criteria for hazardous drinking.

Figures 1 and 2 shows the ROC curves for the AUDIT by using DSM-IV criteria and Figure 3 by using WHO criteria for hazardous drinking for males and females separately. It was associated with AUCs of 0.80 or higher when using either DSM-IV as the gold standard or WHO criteria, suggesting that they performed well as screening measure. The AUC for dependence (Figure 1) was 0.93 (95% CI: 0.90- 0.95) for males and 0.94 (95% CI: 0.92- 0.96) for females whereas for alcohol dependence / abuse (Figure 2) it was 0.99 (95% CI: 0.97- 0.99) for males and 0.97 (95% CI: 0.96- 0.99) for females. The AUC (Figure 3) for hazardous drinkers was 0.80 (95% CI: 0.76- 0.84) for males and 0.89 (95% CI: 0.86- 0.92) for females. Tables 2 and 3 shows the different cut-off values of sensitivity and specificity according to the criteria used. For dependence, results suggest optimal cut-offs ≥11 for both men and women (males: sensitivity 92.7, specificity 84.4, PPV 76.3, NPV 95.5. Females: sensitivity 89.4, specificity 90.5, PPV 72.1, NPV 96.9). For all alcohol use disorders (alcohol dependence/abuse), the best cut-off score was ≥9 for both genders, males (sensitivity 96.7%, specificity 91.7%, PPV 90.3% and NPV 97.2%) and females (sensitivity 94.3%, specificity 91.4%, PPV 80.1% and NPV 97.8%) For hazardous drinking, the most efficient cut-off values are ≥5 for males and ≥4 for females with sensitivity of 93.7 and 91.5 respectively.

**Table 2:** Diagnostic properties of AUDIT for alcohol dependence and alcohol abuse/ dependence

	**AUDIT Score**	**Sensitivity**	**Specificity**	**PPV**	**NPV**
Dependence Males (n=544)	≥9	97.4	79.6	72.1	98.3
	≥10	94.8	81.3	73.3	96.6
	≥11	92.7	84.4	76.3	95.5
	≥12	90.1	85.3	76.8	94.1
	≥13	86.9	87.3	78.7	92.5
	≥14	82.2	88.7	79.7	90.2
	≥15	75.4	89.2	79.1	87.0
Dependence Females (n=524)	≥9	93.8	85.4	63.9	98.0
	≥10	91.2	87.3	66.5	97.3
	≥11	89.4	90.5	72.1	96.9
	≥12	85.0	91.7	73.8	95.7
	≥13	81.4	92.7	75.4	94.8
	≥14	73.5	93.7	76.1	92.8
	≥15	66.4	94.6	77.3	91.1
Alcohol abuse/ dependence Males (n= 544)	≥4	99.8	38.6	56.4	99.8
	≥5	99.6	55.8	64.2	99.4
	≥6	99.6	67.0	70.6	99.5
	≥7	98.8	83.5	82.6	98.8
	≥8	97.5	87.8	86.4	97.8
	≥9	96.7	91.7	90.3	97.2
	≥10	93.4	92.7	91.1	94.6
	≥11	91.7	96.4	95.3	93.6
	≥12	89.2	97.0	96.0	91.9
	≥13	85.1	98.0	97.2	89.2
	≥14	80.5	99.0	98.5	86.5
	≥15	74.3	99.0	98.4	82.9
Alcohol abuse/ dependence Females (n= 524)	≥4	99.3	41.0	38.3	99.4
	≥5	98.6	59.3	47.1	99.1
	≥6	98.6	68.1	53.3	99.2
	≥7	97.9	83.0	68.0	99.1
	≥8	97.2	86.7	72.9	98.8
	≥9	94.3	91.4	80.1	97.8
	≥10	92.2	93.5	83.9	97.0
	≥11	87.9	95.8	88.6	95.6
	≥12	83.0	96.6	90.0	93.9
	≥13	79.4	97.4	91.8	92.8
	≥14	73.0	98.4	94.5	90.8
	≥15	65.2	98.7	94.8	88.5

**Table 3:** Diagnostic properties of AUDIT according to alcohol intake in standard units per week

	**Audit Score**	**Sensitivity**	**Specificity**	**PPV**	**NPV**
Males (n= 544), Alcohol intake 21units/week	4	95.9	41.8	65.8	89.7
	5	93.7	53.4	68.7	78.8
	6	82.9	61.4	71.5	75.5
	7	73.7	71.3	75.0	69.9
	8	71.7	75.3	77.2	69.5
	9	68.9	77.7	78.3	68.2
Females (n= 524),Alcohol intake 14 units / week	4	91.5	70.1	85.0	81.6
	5	75.0	78.3	86.4	62.9
	6	69.7	87.0	90.8	60.8
	7	55.6	92.4	93.1	53.0
	8	52.4	94.6	94.7	51.8
	9	46.8	96.2	95.8	49.4

## Discussion

The first diagnostic evaluation and validation of the AUDIT for alcohol dependence, alcohol dependence/abuse and hazardous drinking in Nepalese language has been reported here. The feasibility of using the AUDIT in the busy out-patient setting by successfully screening 1068 patients has been demonstrated. The prevalence of alcohol dependence and alcohol abuse/ dependence was 32.2% and 40.5% respectively. The prevalence of alcohol dependence was very high in this study. Several epidemiological studies have estimated a higher prevalence of AUDs in medical settings than in the general population [26,27]. Namely, lifetime prevalence of alcohol abuse or dependence has been identified in ~10% of the general population and in 16–36% of outpatients [27]. In a previous study using the CAGE questionnaire Jhingan et al. reported the prevalence of alcohol dependence in Nepal to be 25.8% [5].

AUDIT has demonstrated a high degree of internal consistency over a broad range of diverse settings. In a reliability generalization analysis of studies that appeared in 2000 or before, Shields and Caruso calculated a median reliability coefficient of 0.81, ranging from 0.59 to 0.91 [28]. In a more recent review of 18 studies published since 2002, Reinert and Allen found comparable results with a median reliability coefficient of 0.83, ranging from 0.75 to 0.97 [29]. The Cronbach’s coefficient of 0.82 found in this study is therefore consistent with previous findings.

Although cultural differences may have significantly influenced the cut-off points, it is confirmed by the current study that the Nepali version of AUDIT can be used as a reliable and valid screening tool for alcohol dependence and alcohol dependence/abuse. However the diagnostic performance for identifying hazardous drinking is weaker. The best diagnostic cut-off score for dependence was ≥11 in both males and females, with high NPV (>95% in both sexes) and moderate PPV (76.3% in males and 72.1% in females). Sensitivity and specificity estimates were comparable to those found in studies done in Switzerland where the diagnostic cut-off scores for dependence ranged from 10 to 13 [30]. Tsai et al. reported the same cut-off score (≥11) for diagnosing alcohol dependence among hospitalized Chinese patients [31]. Guo et al. performed an epidemiological survey in a Tibetan population and found AUDIT cut-off scores of 10 and 13 as best diagnostic discriminators for diagnosing alcohol abuse and alcohol dependence, respectively, with sensitivity and specificity estimates >0.84 [21]. Gache et al. conducted a cross-sectional study in three French speaking areas and recommended a cut-off score of ≥13 for the detection of alcohol dependence [18]. These findings are in contrast to the recommendations of the developers of AUDIT, who had set cut-off scores >19 for identifying alcohol dependence [32].

In this study, for alcohol abuse/ dependence the best cut off value was ≥9 for both males and females. Similar values (≥10 for alcohol dependence/abuse) were found by Guo et al. using the Chinese version of the AUDIT in Tibet [21]. However these values are higher than found by Gache et al. in the French version where they found cut-off values of ≥6 for men and ≥5 for women [18]. The sensitivity of the AUDIT was much higher in Nepal (96.7% for males and 94.37% for females) than in France (76.7% for males and 78.7% for females though the specificity was found to be similar.

Diagnostic studies focusing on hazardous drinking are barely comparable because the criteria used to define hazardous drinking vary considerably. For example, the SDU amounts to 8g of pure ethanol in United Kingdom and 14g in the USA. In this study, we used the WHO definition of 10g ethanol per SDU. The diagnostic performance of the AUDIT for hazardous drinking is shown by AUC in Figure 3. The best cut-off score was ≥5 for males and ≥4 for females. For AUDIT, the reported cut-off for hazardous drinking has ranged from ≥4 in a family practice center [33] to ≥10 in hospital in-patients and out-patients who volunteered for the study [34] with other values in between these extremes. Other studies recommended cut-off scores below the standard value of 8 to screen for alcohol-problems of lower intensity than alcohol dependence or abuse. Three of these investigations, which were conducted in primary care or general practice settings determined that the best cut-off scores to identify both hazardous and harmful use was 5 in women and 5–7 in men with sensitivity and specificity estimates ranging from 73 to 96% and 88 to 96%, respectively [18,35,36]. In a general population sample, Rumpf et al. proposed the use of a cut-off score of 5 as optimal for identifying hazardous drinking (sensitivity: 77%; specificity: 80%) [37]. A slightly higher cut-off score of 6 was suggested by Kokotailo et al. for detecting hazardous drinking among U.S. college students (sensitivity: 91%; specificity: 60%) [38].

According to findings of this study the following AUDIT scores can be recommended. For males: 0–4 no problem, 5–10 hazardous drinking, ≥11 alcohol dependence. For females: 0–3 no problem, 4–10 hazardous drinking, ≥11 alcohol dependence.

This study has some limitations. First, the external validity of the findings is limited since our study patients were attending an out-patient department of a university hospital. Secondly, patients may under- or over-report alcohol consumption. Miss-reporting was minimized by including all types of glasses and containers used locally for consumption of alcoholic beverages, and by asking patients several times about the exact frequency and volume consumed., Third, our study population consisted of out-patients attending a tertiary-level care hospital in Nepal, so the results may differ somewhat in other clinical settings like primary care centre and community hospitals. Despite these limitations, this study shows that the use of Nepali version of AUDIT is feasible and can be used reliably in the busy out-patient setting for screening of hazardous drinking and alcohol dependence. Our findings also indicate that commonly used cut-off scores for adults must be lowered when AUDIT is used among the Nepalese population as compared to the cut-off points of ≥19 for alcohol dependence and 8–15 for hazardous drinking recommended by the developers of AUDIT [30].

## Conclusions

This study reveals high prevalence of alcohol use disorders and hazardous drinking in Nepal. The Nepali version of AUDIT can be used as a reliable and valid tool for early detection and diagnosis of individuals with AUDs in a busy clinical setting with a cut-off of ≥11 for alcohol dependence, ≥9 for all alcohol use disorders for both genders. For hazardous drinking, a cut-off score of ≥5 and ≥4 are recommended for males and females respectively. It is clear that there is an urgent need to formulate a policy for alcohol abuse in the country, taking into account the findings of this study.

## Competing interests

The other authors have no competing interests to declare.

## Authors' contributions

BP led the study design, data collection, analysis and drafting of the manuscript. FC, PG and AH participated in analysis, interpretation, and manuscript revisions. DDB participated in data analysis and provided statistical consultation.PK and SR participated in data collection and manuscript revision. All authors read and approved the final manuscript
